# Hospital-onset COVID-19: Associations with population- and hospital-level measures to guide infection prevention efforts

**DOI:** 10.1017/ash.2023.219

**Published:** 2023-09-29

**Authors:** Emily Schmitz, Laura Anderson, Gabrielle Hatas, Marin Schweizer, Nasia Safdar, Daniel Shirley

## Abstract

**Objective:** To characterize hospital-onset COVID-19 cases and to investigate the associations between these rates and population and hospital-level rates including trends in healthcare worker infections (HCW), community cases, and COVID-19 wastewater data. **Design:** Retrospective cohort study from January 1, 2021, to November 23, 2022. **Setting:** This study was conducted at a 589-bed urban Midwestern tertiary-care hospital system. **Participants and interventions:** The infection prevention team reviewed the electronic medical records (EMR) of patients who were admitted for >48 hours and subsequently tested positive for SARS-CoV-2 to determine whether COVID-19 was likely to be hospital-onset illness. Each case was further categorized as definite, probable, or possible based on viral sequencing, caregiver tracing analysis, symptoms, and cycle threshold values. Patients were excluded if there was a known exposure prior to admission. Clinical data including vaccination status were collected from the EMR. HCW case data were collected via our institution’s employee health services. Community cases and wastewater data were collected via the Wisconsin Department of Health Services database. Additionally, we evaluated the timing of changes in infection prevention guidance such as visitor restrictions. **Results:** In total, 156 patients met criteria for hospital-onset COVID-19. Overall, 6% of cases were categorized as definite, 24% were probable, and 70% were possible hospital-onset illness. Most patients were tested prior to a procedure (31%), for new symptoms (30%), and for discharge planning (30%). Also, 53% were symptomatic and 41% received treatment for their COVID-19. Overall, 38% of patients were immunocompromised and 27% were unvaccinated. Overall, 12% of patients died within 1 month of their positive SARS-CoV-2 test, and 11% required ICU admission during their hospital stay. Hospital-onset COVID-19 increased in fall of 2022. Specifically, October 2022 had 16 cases, whereas fall of 2021 (September–November) only had 3 cases total. Finally, similar peaks were observed in total cases by week between healthcare workers, county cases, and COVID-19 wastewater levels. These peaks correspond with the SARS-CoV-2 delta and omicron variant surges, respectively. **Conclusions:** Hospital-onset cases followed similar trends as population and hospital-level data throughout the study period. However, hospital-onset rate did not correlate as strongly in the second half of 2022 when cases were disproportionately high. Given that hospital-onset cases can result in significant morbidity, continued enhanced infection prevention efforts and low threshold for testing are warranted in the inpatient environment.

**Figure:**
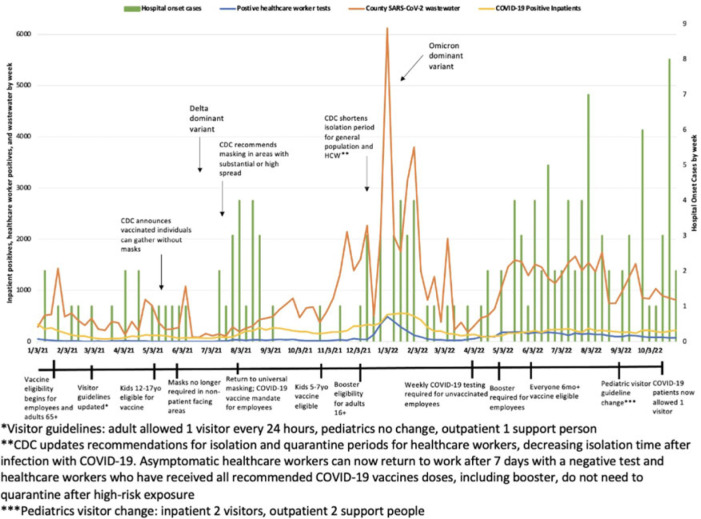

**Disclosure:** None

